# Antifatigue Activity of Glycoprotein from *Schisandra chinensis* Functions by Reducing Oxidative Stress

**DOI:** 10.1155/2020/4231340

**Published:** 2020-07-29

**Authors:** Shuai Shao, Ming-xing Wang, Hong-yin Zhang, Lin Fan, Rong-xin Han, Ying-xin Shen, Ming-ming Yan, Da-qing Zhao

**Affiliations:** ^1^Changchun University of Chinese Medicine, Changchun, Jilin 130117, China; ^2^Jilin Provincial Science and Technology Innovation Center of Health Food of Chinese Medicine, Changchun University of Chinese Medicine, Changchun, Jilin, China; ^3^Research Center of Traditional Chinese Medicine, Affiliated Hospital, Changchun University of Chinese Medicine, Changchun 130021, China

## Abstract

The glycoprotein from *Schisandra chinensis* was obtained with alkali extraction and acid precipitation, purified with DEAE Sepharose Fast Flow and Superdex G-75 column. The molecular composition structure and antifatigue activities of glycoprotein were studied. SCGP's molecular weight was approximately 10 KDa, and it consisted of a carbohydrate component (52.94%) and protein component (47.06%). SCGP comprised mannose, galactoside, rhamnose, glucose, galactose, xylose, arabinose, and fucose, its molar ratio was 2.14 : 1.43 : 1.59 : 8.17 : 8.99 : 3.18 : 18.51 : 1, and it contained 16 kinds of amino acids. SCGP could obviously extend the swimming time in mice by increasing LDH, SOD level, GSH-Px activity, and liver glycogen and decreasing the contents of BUN and MDA. The antioxidant activity of SCGP is a potential mechanism of its antifatigue effect. In vitro antioxidant test showed that SCGP scavenged DPPH and OH radicals in a dose-dependent manner (IC_50_ was 0.91 mg/ml and 0.72 mg/ml).

## 1. Introduction

Fatigue refers to a physiological state in which the body cannot maintain a certain level of function or an organ cannot maintain a predetermined exercise intensity. Long-term fatigue is a subhealthy state, which can make systems of the human body function at low levels, render people more susceptible to physiological or psychological diseases, and has a great negative impact on work efficiency and quality of life [1]. Natural antifatigue preparations such as ginseng, pilose antler, and *Schisandra chinensis* mainly nourish qi and blood with less side effects than that of Western medicine; the antifatigue effect of these compounds is not necessarily smaller than their Western medicine counterparts. Their antifatigue effect is not based on substances, but on regulating metabolism and neuromuscular function. Therefore, the development of natural antifatigue agents has great prospects [2].

In the past few decades, researchers have done a lot of study and formulated various theories on the mechanism of physical fatigue. The most common among them are the theory of energy depletion, instability of the internal environment, and free radical activity. In long-term work or exercise, the first use of energy materials such as glucose and glycogen may be exhausted, leading to the production and accumulation of metabolites in the body [3]. Free radicals are also intermediate metabolites of many important biochemical reactions in the body. After intense exercise, the balance between oxidation and the antioxidant system is destroyed, resulting in excessive production of free radicals and reactive oxygen species (ROS) in various tissues of the body, which may exceed the scavenging capacity of the body's own antioxidant defense system (including enzymes and nonenzymes) and cause oxidative stress. Excessive free radicals and ROS can attack cell macromolecules, which can deteriorate normal cell functions and lead to fatigue due to tissue damage and decreased muscle contractility [4]. Recent studies have confirmed that exogenous antioxidants can reduce oxidative stress and delay physical fatigue by inhibiting or preventing oxidation of oxidizable substrates in cells, inhibiting lipid peroxidation, directly scavenging free radicals, or forming synergistic antioxidant networks with endogenous antioxidants. Therefore, supplementation of exogenous antioxidants to the body is an effective way to delay physical fatigue [5]. In recent years, some natural antioxidant components, such as polysaccharides, saponins, flavonoids [6], and small molecular peptides [7], have been found to have significant antifatigue effects.

*Schisandra chinensis (Turcz.) Baill* has a long history of usage as a tonic and astringent agent in China, Korea, and Japan. According to reports, *Schisandra* has extensive pharmacological functions, including antihepatotoxic [8], antioxidative [9], anticancer, and anti-HIV effects [10], hepatitis, and cancer. At present, studies have revealed that the antifatigue components of *Schisandra* are polysaccharides, lignans [11], and polyphenols, but there are no reports about glycoproteins. Therefore, in this study, the water-soluble glycoprotein from *Schisandra chinensis* (SCGP) was obtained by extraction, the antifatigue and antioxidant activities were investigated, and the possible underlying mechanism of SCGP in mice was explored.

## 2. Materials and Methods

### 2.1. Materials and Chemicals

*Schisandra chinensis* was collected in Changbai Mountain, Jilin Province. It was identified as the dry and mature fruit of *Schisandra chinensis*, a Magnoliaceae plant, by Professor Dacheng Jiang, School of medicine, Changchun University of traditional Chinese medicine. LDH, SOD, GSH-Px, BUN, MDA, and protein were measured by Elisa kits (Nanjing, China). Folin–Ciocalteu's phenol reagent, DPPH (1,1-diphenyl-2-picrylhydrazyl) was purchased from Sigma-Aldrich (Steinheim, Germany). All other chemicals were of analytical grade and were purchased from Beijing Chemical Co.

### 2.2. Extraction and Isolation of SCGP

Dried fruits of *Schisandra chinensis* were homogenized with distilled water (the ratio of sample to solvent is 1 : 35 w/v). The homogenate was adjusted to pH 9.5 with 1 M NaOH and incubated at 35°C for three hours before centrifugation. The supernatant was adjusted to the isoelectric point with 1 M HCl while stirring constantly and held for one hour. After centrifugation, the precipitate was dissolved in water and then dialyzed with distilled water. Then, extract was obtained and applied to a DEAE column (2.6 cm × 30 cm), with sequential elution of 0-1.0 M NaCl and 20 mM Tris-HCl. The protein concentration was determined by Elisa kit. The content of carbohydrate in eluent was determined by the phenol-sulfuric acid method. Three elution peaks were collected separately. The peak 2 fraction proved to have the highest antioxidant activity. Then, peak 2 was purified by Superdex G-75 column (2.6 × 60 cm). The elution peak was spray-dried to obtain SCGP powders.

### 2.3. Basic Properties Analysis

#### 2.3.1. Molecular Mass Determination Using SDS-PAGE

The SCGP sample was separated in an electrophoresis cell, and superlow-molecular-weight markers from Bio-Rad were used [12].The molecular weight of SCGP was determined by comparing the electrophoretic mobility of SCGP with that of molecular weight-labeled proteins (Amersham Biosciences, Sweden) [13].

#### 2.3.2. Chemical Constituents and Contents of SCGP

The protein content of SCGP was quantified by the Bradford method [14]. Amino acid of SCGP was determined using the automatic amino acid analyzer. The SCGP sample was hydrolyzed under vacuum in 6 M HCl at 110°C for 24 h. The hydrolyses were evaporated, and the dried residue was redissolved in 0.02 M HCl. The amount of each amino acid was expressed in percentage of amino acid per 100 g of amino acid [15].

The carbohydrate content of SCGP was determined by the phenol-sulfuric acid method with glucose as reference [16]. The monosaccharide composition was determined by precolumn derivatization with slight revision. The monosaccharide composition was analyzed by HPLC system (USA, Agilent 1100) equipped with a Unitary C 18 column (4.6 *µ*m × 250 mm). The PMP derivative was injected and then gradient eluted with PBS and acetonitrile. The wavelength for UV detection was 250 nm [17].

### 2.4. In Vivo Antifatigue Effect of SCGP

#### 2.4.1. Groups and Treatment

Forty 8-week-old Kunming (KM) mice (18–22 g for each group, 5 males and 5 females) were issued by the Chinese government health and safety certificate (Sheng Chan Xu Ke number SCXK 2018-0014). The mice were then randomly divided into four groups, each with a similar weight, i.e., a control group and three experimental groups (*n* = 10/group) as follows: high-dose group (SCGP-HG), middle-dose group (SCGP-MG), and low-dose group (SCGP-LG) with 1500 mg/kg, 750 mg/kg, and 375 mg of SCGP administered per kg of body weight, respectively. Animal ethics committee of Changchun University of Chinese medicine approved the mice protocols (CCZYY AEC-022).

#### 2.4.2. Measurement of Weight-Loaded Swimming Capacity

The weight-loaded swimming test was used to evaluate the antifatigue effects of SCGP. In brief, after 30 minutes of the last administration of SCGP, the mice were placed in the standard weight-bearing swimming test swimming pool (the depth of the water was more than 30 cm, the temperature of the water was about 25°C, and 5% of the mouse body weight of the metal block). Then, the time from the beginning of swimming to the death of the mice was recorded.

#### 2.4.3. Measurement of Serum and Liver Glycogen Parameters

After the last gavage, the mice were forced to swim for 90 minutes without loads and then allowed to rest for 30 minutes. Blood was collected via the eyes, and the serums were centrifuged (4000 rpm at 4°C for 15 minutes). Blood urea nitrogen (BUN), lactate dehydrogenase (LDH) in serum, and liver glycogen levels were measured using Elisa kits (Biotechnology Co., Ltd., Shanghai, China).

### 2.5. In Vivo Antioxidant Activities of SCGP

#### 2.5.1. Groups and Treatment

After a week of adaption, the mice were randomly divided into six groups (*n* = 10/group), i.e., control group (CG), model control group (MG), positive control group (PG), high-dose group (SCGP-HG), middle-dose group (SCGP-MG), and low-dose group (SCGP-LG). The aging model was induced by subcutaneous injection of *D*-galactose at the back of the neck once daily for 30 days, and the CG was injected equivalently with saline only.

CG consisted of normal mice injected with 0.5 ml normal saline, MG consisted of aging mice injected with 0.5 ml of normal saline, and PG consisted of aging mice injected with 0.5 ml of 50 mg/kg vitamin C. SCPG-HG consisted of aging mice injected with 0.5 ml of 1500 mg SCGP/kg body weight dissolved in saline, SCGP-MG consisted of aging mice injected with 0.5 ml of 750 mg SCGP/kg body weight dissolved in saline, and SCGP-LG consisted of aging mice injected with 0.5 ml of 375 mg SCGP/kg body weight dissolved in saline. From the 11th day to the 30th day, all injections were delivered intraperitoneally once daily.

#### 2.5.2. Measurement of Antioxidant Enzymes and Lipid Peroxidation in the Liver

The mice were euthanized immediately after the blood was collected. The livers of the mice were then collected and homogenized in 10% solution with normal saline at 4°C. The antioxidant status of supernatant was evaluated. The activities of SOD, GSH-Px, and the content of MDA were determined by commercially available kits (Nanjing Jiancheng Biology Engineering Institute, China) according to the manufacturer's protocol.

### 2.6. In Vitro Antioxidant Activities of SCGP

#### 2.6.1. Measurement of 1,1-Diphenyl-2-picrylhydrazyl Radical Scavenging Activity

The DPPH radical scavenging activity of SCGP was analyzed by the Bersuder method with slight modifications. In brief, 1 ml SCGP at different concentrations was mixed with 3 ml of the newly prepared 0.4 mM DPPH solution. The mixture was kept in the dark for 30 minutes at room temperature, and the absorbance at 517 nm was measured using a UV-visible spectrophotometer (UV-1700, Shimadzu, Japan). Vitamin C was used as a positive reference. All experiments were in triplicate [18]. The ability to scavenge DPPH radicals was calculated by the following formula:

(1) $$\begin{array}{c} \text{scavenging }\mathrm{rate}\;\left(\%\right)=1-\left[\frac{\left(A_{1}-A_{2}\right)}{A_{0}}\right]\times 100, \end{array}$$

where *A*_0_ is the absorbance of the control (blank, without sample), *A*_1_ is the absorbance of the sample, and *A*_2_ is the absorbance of VC.

#### 2.6.2. Measurement of Hydroxyl Radical Scavenging Activity

The scavenging activity of hydroxyl radical by SCGP was carried out using the Li method with minor changes. In brief, different concentrations of SCGP were mixed with 0.2 M phosphate buffer (pH 7.5), 0.75 mM 1, 10-phenanthroline, 0.75 mM FeSO_4_, and 0.01% H_2_O_2_. The mixture was reacted at 37°C in a water bath for 30 minutes, and then, its absorbance at 536 nm was determined against a blank using a UV-visible spectrophotometer (UV-1700, Shimadzu, Japan). Vc was used as a positive reference. All experiments were in triplicate [19]. The ability to scavenge the hydroxyl radical was calculated using the following equation:

(2) $$\begin{array}{c} \text{scavenging rate }\left(\%\right)=\left[\frac{\left(A_{2}-A_{3}-A_{1}\right)}{\left(A_{0}-A_{1}\right)}\right]\times 100, \end{array}$$

where *A*_0_ is the absorbance of control (blank, without sample), *A*_1_ is the absorbance of the negative control, *A*_2_ is the absorbance of the sample, and *A*_3_ is the absorbance of *Vc*.

## 3. Results

### 3.1. Purification and Characterization of SCGP

**Figure 1:**
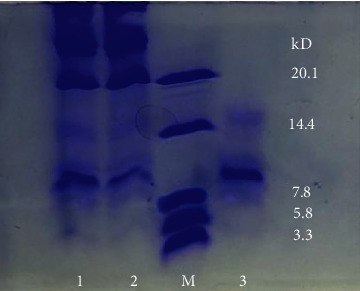
Determination of molecular weight of SCGP by SDS-PAGE. 1 and 2: glycoprotein extract; M: superlow-molecular-protein standard; 3: purified glycoprotein (SCGP).

SCGP was isolated and purified as described previously. As shown in Figure 1, SDS-PAGE showed SCGP is the glycoprotein with the molecular weight of approximately 10 kDa. The carbohydrate and protein contents of SCGP were about 52.94% and 47.06%.

### 3.2. Chemical Composition of SCGP

As shown in Table 1, SCGP contains 16 kinds of amino acids; among them, seven are essential amino acids including phenylalanine, isoleucine, lysine, methionine, threonine, proline, and leucine, accounting for 31.28% of the total amino acids. SCGP is comprised of mannose, galactoside, rhamnose, glucose, galactose, xylose, arabinose, and fucose (Table 2. The molar ratio of each monosaccharide was 2.14 to 1.43 to 1.59 to 8.17 to 8.99 to 3.18 to 18.51 to 1.

### 3.3. Effects on Body Weight and Weight-Loaded Swimming Capacity

The results show that, compared with the CG, there were no significant differences in the initial weights and final weights of the SCGP groups (*P* > 0.05). Therefore, after SCGP treatment, the weight of mice did not change significantly (Table 3). As shown in Table 3, the weight-loaded swimming times in the CG, SCGP-LG, SCGP-MG, and SCGP-HG were 15.70, 19.90, 26.80, and 33.30 minutes, respectively. Compared with that of the CG, the swimming times in all three SCGP groups were longer, and the differences were very significant in the SCGP-MG and SCGP-HG (*P* < 0.01); significant differences were observed in the SCGP-LG (*P* < 0.05). Our results showed that SCGP had a significant activity on the weight-loaded swimming times in this experiment.

### 3.4. Effects on Biochemical Serum Parameters and Glycogen Storage

**Figure 2:**
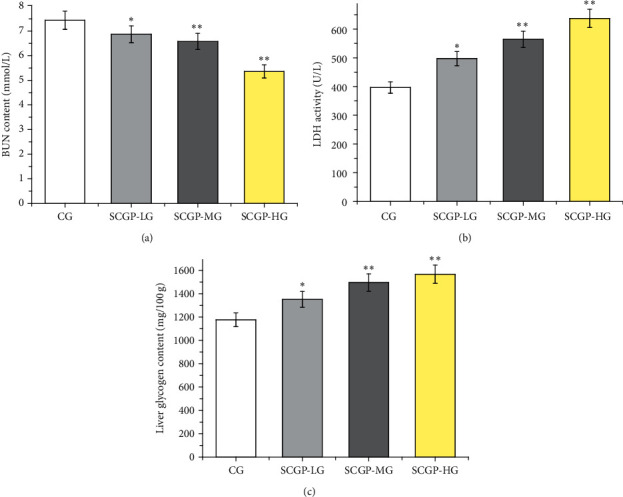
Effect of SCGP on the levels of BUN, (a) LDH, (b) and liver glycogen (c) in mice after swimming. SCGP-LG is the low-dose group of SCGP. SCGP-MG is the middle-dose group of SCGP. SCGP is the high-dose group of SCGP. Results are presented as mean ± SD, *n* = 3. ^*∗*^*P* < 0.05 and ^*∗∗*^*P* < 0.01 compared to the control group.

As shown in Figure 2, the level of BUN in three SCGP groups was significantly reduced when compared to the control group (*P* < 0.05) for the low-dose and middle-dose groups and *P* < 0.01 for the high-dose group, and the LDH activity was very significantly increased in the middle-dose and high-dose groups (*P* < 0.01). Liver glycogen is another index of fatigue. Compared with that of the control group, the liver glycogen levels in all three SCGP groups were increased, and the difference was very significant in the middle-dose and high-dose groups (*P* < 0.01); significant differences in the low-dose group were also observed (*P* < 0.05).

### 3.5. Effects on Antioxidant Enzymes and Lipid Peroxidation

**Figure 3:**
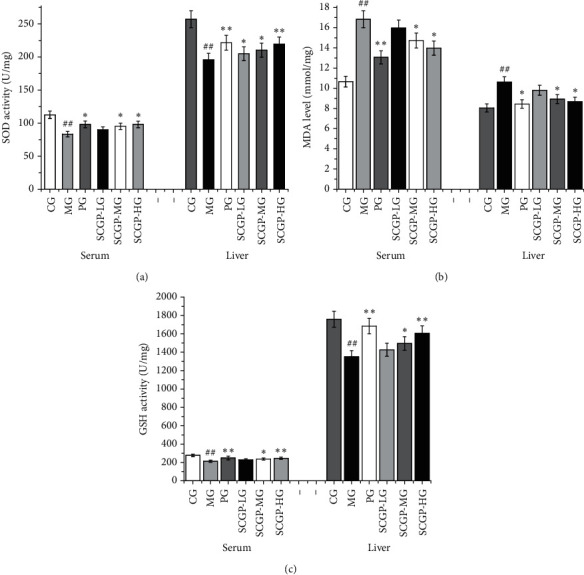
The levels of SOD, (a) MDA, (b) and GSH (c) of mice in serum and liver. SCGP-LG is the low-dose group of SCGP. SCGP-MG is the middle-dose group of SCGP. SCGP is the high-dose group of SCGP. Data are expressed as mean ± SD, *n* = 3. ^*∗*^*P* < 0.05 and ^*∗∗*^*P* < 0.01 compared to the control group; ^#^*P* < 0.05 and ^##^*P* < 0.01 vs. the model group.

As shown in Figure 3(a), SOD activities of the MG were decreased when compared to the CG. The SOD activities in the PG, SCGP-LG, SCGP-MG, and SCGP-HG were increased in the serum and liver. Moreover, SOD activities in the livers of the SCGP-HG were extremely significantly different than the MG (*P* < 0.01). The MDA content of the MG was significantly more increased than the CG, both in the serum and liver (Figure 3(b)). Compared with the MG, the content of MDA in the serum and liver was decreased in the three dosage groups, and SCGP-MG and SCGP-HG were significant. The GSH-Px levels of the MG were reduced significantly compared to the CG (Figure 3(c)). Compared with the MG, GSH-Px activity in the PG, SCGP-LG, SCGP-MG, and SCGP-HG was increased in the serum and liver. Moreover, SOD activities in the livers of the SCGP-HG were extremely significantly different from the MG.

### 3.6. Effects on DPPH Radical and Hydroxyl Radical Scavenging Activity

**Figure 4:**
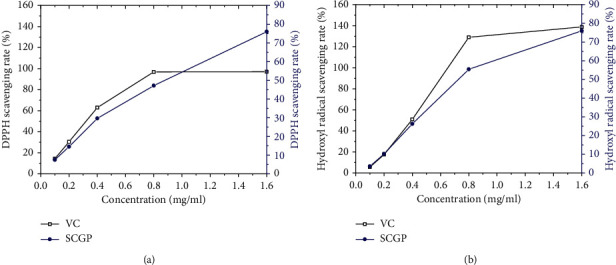
(a) DPPH scavenging rate of SCGP; (b) hydroxyl radical scavenging rate of SCGP.

Our results showed the maximum elimination rate of SCGP was nearly 80% of DPPH in comparison with VC (Figure 4(a)). In addition, the activity of DPPH inhibition was dose-dependent. When the concentration was 0.138 to 2.2 mg/ml, the scavenging rate of DPPH by SCGP increased along with the increase in mass concentration. As shown in Figure 4(b), when the concentration was 0.136 to 2.17 mg/ml, the percentage of scavenging SCGP ranged from 2.239% to 61.94%, and the scavenging abilities of VC ranged from 2.273% to 137.879%. These results showed scavenging of hydroxyl radicals in a dose-dependent manner.

## 4. Discussion

**Figure 5:**
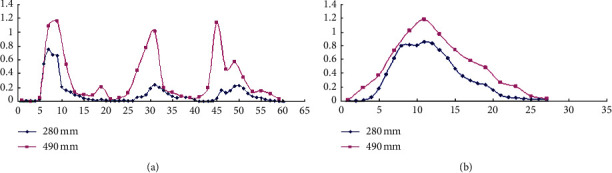
(a) Elution peaks of SCGP from DEAE-Sepharose Fast Flow; (b) elution peaks of SCGP from Sephadex G-75.

In this study, by comparing different extraction methods, alkali extraction and acid precipitation were determined to SCGP. Then, the central combination Box–Behnken method was used to optimize the extraction process of SCGP. The optimum extraction process was determined as the pH value of alkali extract 9.5, the extraction temperature 35°C, and the extraction time 3 h. SCGP was purified by DEAE and Superdex G-75 column (Figure 5).

In the study of pharmacodynamics, antifatigue and antioxidation experiments were designed. The results indicate that SCGP could significantly extend the weight-loaded swimming time, increase LDH, SOD activity, GSH-Px activity, and liver glycogen, and simultaneously decrease the contents of BUN and MDA. The antifatigue effect may be associated with the inhibition of oxidative stress. It is concluded that SCGP plays an important role in improving the body's adaptability to exercise load, improving exercise endurance, and eliminating metabolic waste rapidly.

The weight-loaded swimming test is the most commonly used experimental model for evaluation of antifatigue activities, and swimming time is used as an important indicator to reflect exercise endurance. Sugar is an important energy substance in the body, which is mainly stored in the form of muscle glycogen and liver glycogen. Physical activity mainly depends on glycogen to obtain energy. When glycogen is consumed in large amount, the body's ability to move was reduced. Therefore, glycogen reserve can directly affect the body's exercise capacity, and reserves can improve exercise capacity, thereby delaying the occurrence of exercise fatigue [20]. Along with the increasing quantity of exercise, the body cannot get enough energy from carbohydrates and fats, and then proteins and amino acids have a stronger catabolism to compensate for energy consumption. Being a metabolite of proteins and amino acids, urea nitrogen is used as a representative index to reflect the degree of fatigue [21]. There is a remarkable correlation between the level of BUN and degree of fatigue. At the same time, due to adequate oxygen supply, anaerobic breathing is carried out to provide energy. Lactic acid and H^+^ accumulation from glycolysis resulted in the decrease in pH level, which was also the cause of fatigue [22].

In addition, strenuous exercise can lead to imbalance between oxidation and antioxidant systems in vivo and produce a large number of oxygen free radicals, which cause oxidative stress. There is a lot of evidence that ROS is the cause of protein oxidation induced by exercise and has a strong effect on muscle fatigue [23]. MDA is the main metabolite of lipid peroxidation formed by oxygen free radicals attacking polyunsaturated fatty acids in biofilm. The content of MDA reflects not only the formation of oxygen free radicals and the intensity of lipid peroxidation in vivo but also the level of tissue damage. MDA is a sensitive indicator of the body's free radical metabolism, which can objectively reflect the level of free radicals produced by the body [24]. On the contrary, SOD is an important antioxidant enzyme in the organism, which plays an important role in the oxidation and antioxidant balance of the body. If the activity of SOD in vivo decreased, it can cause lipid peroxidation and lead to cell dysfunction [25]. Furthermore, GSH-Px plays a crucial role against oxidative stress, which can turn toxic substances into innocuous products by scavenging the free radicals. Hence, adequate GSH-Px is essential for preventing oxidative damage [26]. In this study, SCGP can significantly increase SOD and GSH-Px activity and decrease the contents of MDA, and these findings strongly indicate SCGP was able to protect the cells from lipid oxidant.

To test whether SCGP has a direct antioxidative effect, we also performed the in vitro DPPH and hydroxyl radical scavenging test. These methods are commonly utilized to evaluate the radical scavenging activity of plant extracts. In this study, SCGP possessed pronounced scavenging activity against DPPH and hydroxyl radicals.

## 5. Conclusion

A novel glycoprotein (SCGP) with antifatigue and antioxidant activity was isolated from *S. Chinensis*. The molecular weight of SCGP was approximately 10 KDa, and the monosaccharide and amino acid compositions have been determined. Moreover, SCGP could significantly prolong the weight-loaded swimming time of mice by enhancing LDH, SOD activity, GSH-Px activity, and liver glycogen; it also reduced BUN and MDA levels. The antioxidant activity of SCGP is a potential mechanism of its antifatigue effect. SCGP may be a new natural functional food or agent for antifatigue.

## Figures and Tables

**Table 1:** The amino acid composition of SCGP.

No.	Amino acid	g/100 g
1	Asp	3.16
2	Thr	0.68
3	Ser	1.77
4	Glu	5.96
5	Gly	2.29
6	Ala	2.12
7	Cys	0.2
8	Val	1.24
9	Met	0.27
10	Ile	2.04
11	Leu	1.64
12	Tyr	1.76
13	Phe	2.04
14	Lys	0.93
15	His	0.46
16	Arg	4.72
Total amino acid		31.28

**Table 2:** The monosaccharide composition of SCGP.

Retention time	Methylated sugar	Molar ratio
20.371	Mannose	2.14
21.563	Galactoside	1.43
23.267	Rhamnose	1.59
24.758	Glucose	8.17
28.434	Galactose	8.99
29.691	Xylose	3.18
31.005	Arabinose	18.51
33.270	Fucose	1

**Table 3:** The weight and weight-loaded swimming time of mice in all treatment groups.

Group	Mouse weight (g)		Swimming time (min)
	Initial (g)	Final (g)	
CG	27.60 ± 1.48	38.60 ± 1.75	15.70 ± 4.16
SCGP-LG	28.10 ± 1.51	38.80 ± 2.14	19.90 ± 4.58^*∗*^
SCGP-MG	27.90 ± 1.45	39.10 ± 2.04	26.80 ± 5.87^*∗∗*^
SCGP-HG	27.80 ± 1.47	38.70 ± 1.98	33.30 ± 7.89^∗∗^

*x* ± SD; *n* = 10; ^*∗*^*P* < 0.05 and ^*∗∗*^*P* < 0.01 vs. CG.

## Data Availability

The data used to support the findings of this study are available from the corresponding author upon request.
